# Genome-Wide Analysis of Host Factors in Nodavirus RNA Replication

**DOI:** 10.1371/journal.pone.0095799

**Published:** 2014-04-21

**Authors:** Linhui Hao, Brett Lindenbach, Xiaofeng Wang, Billy Dye, David Kushner, Qiuling He, Michael Newton, Paul Ahlquist

**Affiliations:** 1 Institute for Molecular Virology, University of Wisconsin-Madison, Madison, Wisconsin, United States of America; 2 Howard Hughes Medical Institute, University of Wisconsin-Madison, Madison, Wisconsin, United States of America; 3 Department of Statistics, University of Wisconsin-Madison, Madison, Wisconsin, United States of America; 4 Department of Biostatistics and Medical Informatics, University of Wisconsin-Madison, Madison, Wisconsin, United States of America; 5 Morgridge Institute for Research, University of Wisconsin-Madison, Madison, Wisconsin, United States of America; The Ohio State University, United States of America

## Abstract

Flock House virus (FHV), the best studied of the animal nodaviruses, has been used as a model for positive-strand RNA virus research. As one approach to identify host genes that affect FHV RNA replication, we performed a genome-wide analysis using a yeast single gene deletion library and a modified, reporter gene-expressing FHV derivative. A total of 4,491 yeast deletion mutants were tested for their ability to support FHV replication. Candidates for host genes modulating FHV replication were selected based on the initial genome-wide reporter gene assay and validated in repeated Northern blot assays for their ability to support wild type FHV RNA1 replication. Overall, 65 deletion strains were confirmed to show significant changes in the replication of both FHV genomic RNA1 and sub-genomic RNA3 with a false discovery rate of 5%. Among them, eight genes support FHV replication, since their deletion significantly reduced viral RNA accumulation, while 57 genes limit FHV replication, since their deletion increased FHV RNA accumulation. Of the gene products implicated in affecting FHV replication, three are localized to mitochondria, where FHV RNA replication occurs, 16 normally reside in the nucleus and may have indirect roles in FHV replication, and the remaining 46 are in the cytoplasm, with functions enriched in translation, RNA processing and trafficking.

## Introduction

All viruses depend on host cell functions for multiple replication steps, and modulate host pathways to make infected cells a better environment for virus replication. Accordingly, both targeted and genome-wide studies identifying cellular factors and virus-host interactions that support or inhibit virus replication offer great value for understanding infection and developing novel antiviral approaches[1]–[3].

**Figure 1:**
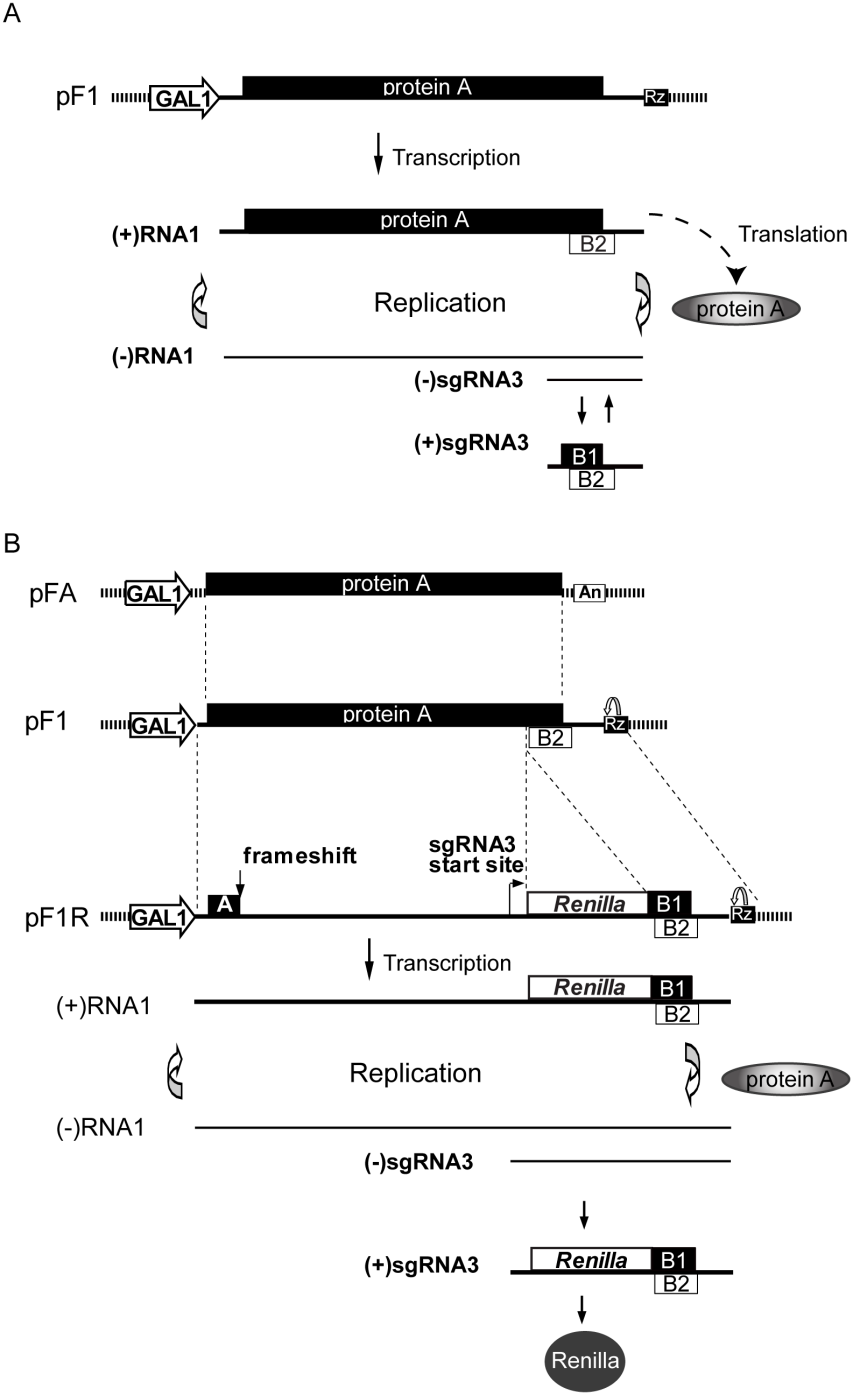
Structure, plasmid-directed synthesis and replication of wt FHV RNA1 and *Renilla* luciferase-expressing derivative F1R. A) Diagram of FHV RNA1 expression plasmid pF1, which uses the galactose-inducible, glucose-repressible *GAL*1 promoter to express a wt FHV RNA1 transcript whose 3′ end is formed by self-cleavage by a hepatitis delta virus ribozyme [48]. RNA1 is translated to produce protein A, the multifunctional FHV RNA synthesis protein, which directs RNA1 replication and, through a truncated negative-strand intermediate ((-) sgRNA3), production and subsequent replication of RNA3 (sgRNA3), a subgenomic mRNA that encodes two ORFs: B1, the C-terminus of protein A, and B2, an RNA silencing suppressor. B). Plasmid pFA is a pF1 derivative that retains the complete FHV protein A ORF but has the FHV 5′ and 3′ untranslated regions, which contain essential cis-acting RNA replication signals, replaced with nonviral sequences. The resulting transcript from the pFA *GAL1* promoter translates full length, wt FHV protein A, but cannot serve as an RNA replication template. Plasmid pF1R is a second pF1 derivative that retains the full length RNA1 sequence, but bears an early frameshift mutation in the protein A ORF and an insertion of the *Renilla* luciferase ORF immediately after the initiation codon of the B1 ORF AUG in RNA3. The resulting pF1R transcript thus cannot translate protein A but, when protein A is provided *in trans* from pFA, is replicated and produces a *Renilla* luciferase-expressing subgenomic RNA3 derivative.

The largest genetic class of viruses are the positive-strand RNA viruses, which encapsidate messenger-sense single-stranded RNA and include many important human pathogens like hepatitis C virus, West Nile virus, many animal pathogens, and the majority of known plant viruses. One model that has been used to analyze the mechanisms of positive-strand RNA virus replication and virus-host interaction is Flock House virus (FHV), the best-studied member of the *Nodaviridae* family of animal viruses. FHV has a small, bipartite RNA genome. The smaller genomic RNA, RNA2 (1.4 kb), encodes the capsid protein precursor. The larger genomic RNA, RNA1 (3.1 kb), encodes multifunctional protein A, which has RNA-dependent RNA polymerase, self-interaction and membrane-interaction domains and is the sole viral protein required for FHV RNA replication [4]–[6]. In vivo, RNA1 can replicate independently of RNA2, by translating protein A, which then produces and copies a negative-strand RNA1 replication intermediate (Figure 1A). Protein A-directed replication of RNA1 also produces a small sub-genomic mRNA, RNA3, containing two overlapping open reading frames (ORFs) encoding proteins B1 and B2. While B1, which corresponds to the C-terminus of protein A, is not required for FHV replication [7], B2 is an RNAi inhibitor required for efficient FHV replication in insect or nematode cells [8], [9]. As with all positive-strand RNA viruses, FHV RNA replication occurs on intracellular membranes. Specifically, in *Drosophila* cells, a natural host for productive FHV infection, FHV RNA replication and transcription occur in virus-induced, ∼50 nm invaginations of outer mitochondrial membranes [10].

To identify host genes that facilitate or inhibit FHV replication, we utilized FHV’s ability to replicate in an unusually wide range of cells. Although originally isolated from insects, FHV RNA also efficiently replicates and directs the production of infectious virions in mammalian cells, plant cells, and the yeast *Saccharomyces cerevisiae,* a widely used model for genetics, cellular and molecular biology [4]. Many powerful genetic resources exist for *S. cerevisiae*, including an ordered, genome-wide, single gene deletion or yeast knockout (YKO) library that allows systematically studying the effects of each gene on a selected process, such as FHV replication. This YKO library was previously used to identify genes that facilitate or inhibit brome mosaic virus (BMV) and tombusvirus RNA replication [11], [12]. While they are all positive-strand RNA viruses, BMV and tombusviruses differ from FHV in many aspects of their cellular and molecular biology and naturally infect plant hosts rather than animal hosts as for FHV and *Nodaviridae*. Moreover, very limited overlap was found among the host genes that strongly modulated BMV and tombusvirus replication. Thus, analysis of host factors required for FHV replication should shed further light on the replication pathways and host interactions of positive-strand RNA viruses.

Here we present a systematic functional genomics analysis of host genes that affect FHV replication using ∼4,500 yeast deletion mutant strains, each with a single known ORF knocked out, covering 80% of the yeast genome. Initial high throughput screening was conducted with an RNA1 derivative containing a *Renilla* luciferase reporter gene, whose expression depends on viral RNA replication and sub-genomic mRNA synthesis. Implicated candidate genes were then validated by further testing of their effects on the replication of wt FHV RNA1, confirming 65 genes whose deletion produced significant effects on FHV genomic RNA1 replication and sub-genomic RNA3 production.

## Results and Discussion

### Generating an FHV RNA1 Derivative for High-throughput Screening

To facilitate informative, high throughput assays of FHV RNA-dependent RNA replication in the ordered YKO strain library, we generated plasmid pF1R, which uses the galactose-inducible *GAL1* promoter to express an FHV genomic RNA1 derivative with two engineered changes (Figure 1B). The first change addressed the complication that wt FHV RNA1 is both the mRNA that expresses RNA replication protein A and an RNA replication template that is multiplied by protein A (Figure 1A). Consequently, any host gene deletion in the YKO library that interfered with FHV RNA replication would have its effects further amplified by the resulting secondary inhibition of protein A expression. To avoid such unwanted amplification of YKO mutant effects, we separated RNA1’s mRNA function from its replication template activity by inserting a frameshift mutation early in the protein A ORF, and provided protein A in trans by transcription of a non-replicating protein A mRNA from a second DNA expression plasmid, pFA (Figure 1B and references [4], [13]).

Second, to facilitate high throughput assays of FHV RNA replication in the ordered YKO strain library, the *Renilla* luciferase gene was inserted immediately after the AUG of B1 ORF in sub-genomic RNA3, such that *Renilla* luciferase activity was expressed in a fashion that depended on and served as a measure of FHV RNA-dependent RNA replication and subgenomic RNA3 synthesis [4] (Figure 1B).

**Figure 2:**
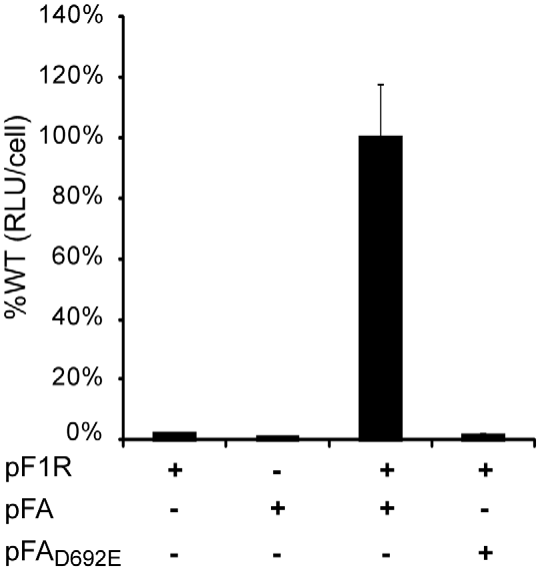
Validation of FHV replication-dependent luciferase expression. Yeasts transformed with the indicated plasmids were assayed for luciferase activity. As shown, significant luciferase activity was only observed when there is active FHV RNA replication and subgenomic mRNA synthesis, which depend on expressing both functional protein A (expressed from pFA) and a functional FHV RNA1 derived template RNA (from pF1R). Neither protein A (pFA) alone, nor the RNA1 derived template RNA (pF1R) alone produced luciferase activity above background. The need for FHV RNA synthesis is further demonstrated by the effects of a polymerase-inactivating mutation in the protein A active site (expressed from pFA(GED)), which reduces luciferase activity to background level even when expressed together with pF1R.

The data in figure 2 confirmed that, as intended, *Renilla* luciferase activity was only detected when both pFA and pF1R were provided (condition 3), as required for RNA1 replication and subgenomic RNA3 production [4], [13]. Only low level background signals were detected if either pFA or pF1R were omitted (conditions 1–2), or if wt pFA was replaced with its nonfunctional derivative pFA(D692E) (condition 4), a protein A mutant with an inactivating single D_692_ to E amino acid change in a conserved polymerase motif [14].

### Testing 4,491 Yeast Knock-Out (YKO) Strains for Effects on FHV Replication

Except for 301 yeast deletion strains with previously annotated significant growth defects, the remaining 4,491 strains of the BY4743 homozygous diploid yeast knock-out (YKO) non-essential library were tested for their ability to support FHV RNA replication as follows: yeasts were transformed with plasmid pFA and pF1R, and the resulting yeast transformants were grown in liquid synthetic defined (SD) medium with histidine and leucine omitted to maintain plasmid selection. Glucose was provided as the carbon source for the first passage in liquid medium. After one day of growth, the yeasts were sub-cultured to a starting density of OD_600_ = 0.075 in SD medium with galactose for two more passes to induce virus replication. Yeast growth was monitored by measuring culture OD_600_. For each strain, *Renilla* luciferase activity was measured using a whole cell assay as described [11], as a measure of FHV-directed RNA replication and expression.

Two independent passes of the above screen were performed across the 4,491 YKO strains, which were arrayed in 48 96-well plates. The YKO library contains 84 duplicate strains, which leaves 4,407 unique yeast deletion mutant strains. In the first and second pass, 204 and 212 unique strains respectively could not be assayed as they either failed to be transformed with pFA + pF1R or to grow in galactose liquid medium. Of the affected strains, 147 were not assayed in either pass, and 122 were only assayed once. The remaining 4,138 unique strains were tested twice for FHV replication, with a small portion tested four times because they were duplicated in the library. The Pearson’s correlation coefficient between the two passes is 0.446.

To stabilize the variance between samples with high and low luciferase activity readings, a power transformation (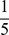 root) was applied to the luciferase readings [15], [16]. Based on the common assumption that most cell genes should not strongly affect virus replication, as was confirmed by the screen results, each reading then was normalized to the median of the relevant 96-well plate. The complete data from both primary screen passes is shown in table S1.

### Validation Testing Confirms 65 Genes with Strong Effects on FHV Replication

The data from the two screening passes of the YKO library were filtered by several criteria to select strains for further analysis. First, experience shows that, irrespective of the host pathways affected, most mutant yeast strains with extreme high or low growth rates show strong, apparently non-specific effects on virus-directed reporter gene expression [11]. In general, FHV-expressed luciferase activity accumulates to lower levels per cell in fast-growing strains, and to higher levels per cell in slow-growing strains. These results appear consistent with effects dominated by changes in the balance between varying host cell division rates and a relatively fixed rate of FHV RNA replication. Thus, strains with extreme changes in growth rate appear less likely to be informative about specific mechanisms of virus replication and virus-host interaction. Accordingly, to avoid mis-calling non-specific effects of cell growth rate as specific effects on virus replication, in each pass the final OD_600_ of each yeast strain before *Renilla* luciferase assay was normalized to the median of its respective 96-well plate as a measure of growth rate, and the strains with the highest 5% and lowest 5% of growth rates from each primary screen pass (see Table S1) were excluded from the next phases of analysis pursued here.

For initial validation testing, we first selected the 79 strains (Table S1) that in both primary screen passes showed >5-fold changes in luciferase expression relative to wild type yeast (Table S1). Next, to provide a more inclusive assay, 180 additional strains (Table S1) that showed at least a two-fold change in FHV-directed luciferase expression in both primary screen passes were re-screened six times using the *Renilla* luciferase assay. T-tests of the resulting data confirmed that, at a false discovery rate (FDR) of ≤5%, 46 strains (25%) showed significant differences from wild type yeast (Table S1).

**Figure 3:**
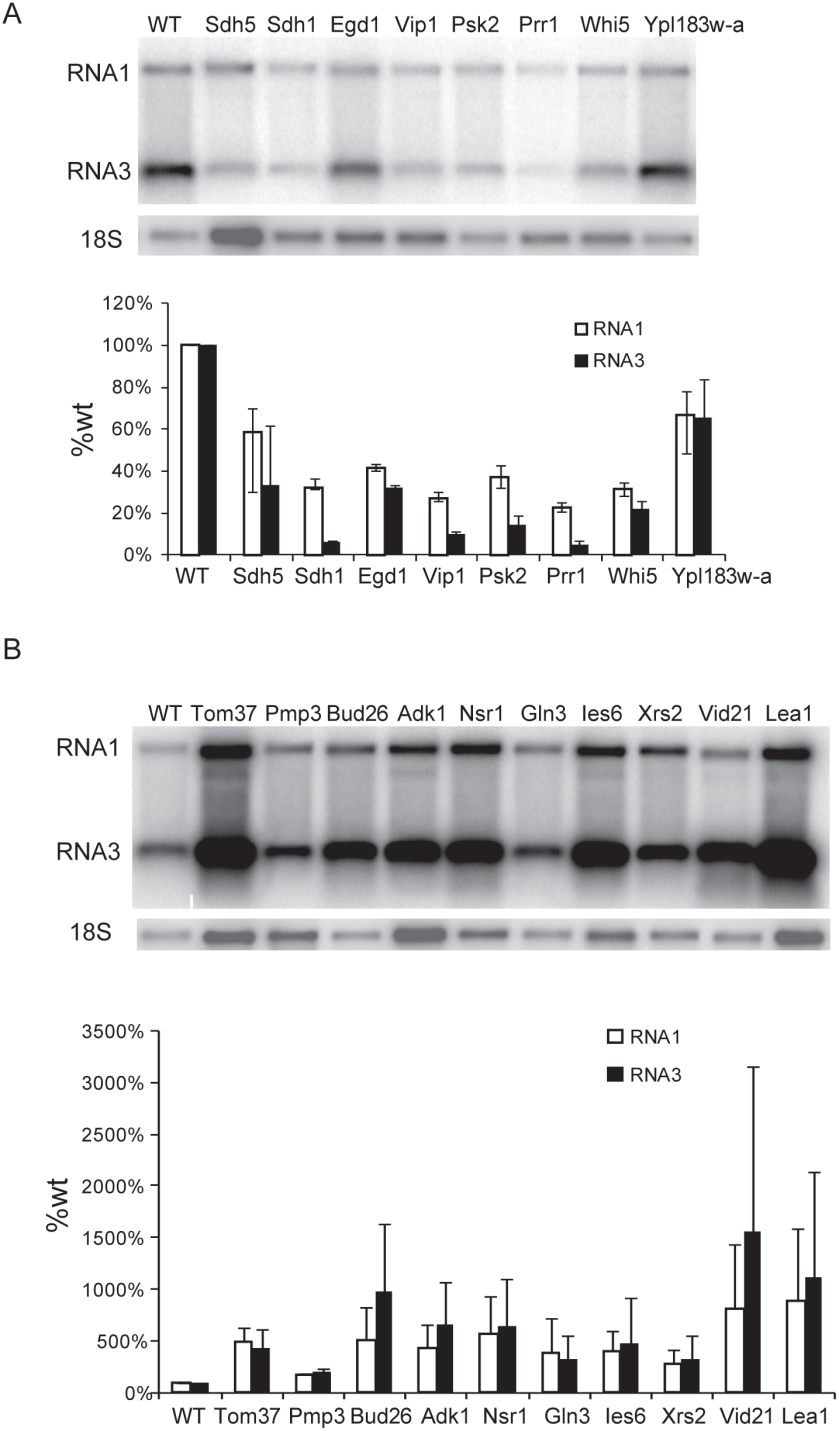
FHV RNA1 replication in selected yeast deletion mutants. Yeast strains with the indicated gene deletions were transformed with plasmid pF1 (Figure 1A) and, following galactose-induction of pF1 transcription, FHV genomic RNA1 replication and subgenomic RNA3 production were assayed by Northern blot hybridization. The histograms show the averages and standard deviations of RNA1 and RNA3 levels across four independent experiments. Representative Northern blots are shown above each histogram. A). FHV wild type RNA1 replication in all 8 deletion mutants whose deleted gene functions facilitate FHV replication. WT denotes the no deletion control. B). FHV wild type RNA1 replication in a selected subsets of yeast deletion mutants whose deleted gene functions normally inhibit FHV replication. Accordingly, these strains show increased FHV replication.

Next, the resulting 79+46 = 125 strains were analyzed for effects on FHV RNA replication using a more direct assay. These strains were transformed with plasmid pF1 (Figure 1B), which expresses wt FHV RNA1 that both expresses protein A and directs its own RNA replication in *cis*. FHV genomic RNA1 and subgenomic RNA3 levels were measured by Northern blotting and normalized to the 18S rRNA level in each sample (Figure 3). Four independent repeats of such Northern blot data for all 125 candidate strains from the primary screens then were analyzed by paired t-tests for changes in both RNA1 and RNA3 accumulation relative to wt yeast. For the 79 mutant strains with >5 fold changes in FHV-directed luciferase expression in the primary screens, these Northern blot assays validated 43 strains (55%) as distinct from wt yeast at a false discovery rate of≤5% (Table S1). Similarly, for the 46 strains with 2- to 5-fold changes in luciferase expression, 22 strains (48%) were validated (Table S1). These validation rates are similar to those found, e.g., in prior screens for host factors that modulate *Drosophila* C virus replication [17].

The 43+22 = 65 confirmed genes, their annotated functions, and average RNA1 and RNA3 accumulation levels are shown in tables 1 and 2. Figure 3 shows sample Northern blots with histograms of RNA1 and RNA 3 levels relative to those of wild type yeast.

**Table 1:** Yeast genes that support FHV replication.

Mitochondrial								
**ORF**	**Gene**	**Molecular Function**	**RNA1 (%WT)**	**t1**	**q1**	**RNA3 (%WT)**	**t3**	**q3**
**Translation**								
YPL183W-A		structural constituent of ribosome	67%	−5.1	1.7%	65%	−3.5	4.5%
Metabolism								
YOL071W	SDH5	oxidoreductase activity	59%	−6.0	1.5%	34%	−3.5	4.4%
YKL148C	SDH1	oxidoreductase activity	32%	−13.8	0.6%	6%	−41.0	0.2%
YPL037C	EGD1	unfolded protein binding	42%	−41.5	0.3%	32%	−44.6	0.2%
**Cytoplasmic**								
**Chaperone**								
YLR410W	VIP1	inositol phosphate kinase	27%	−23.3	0.3%	10%	−34.0	0.2%
**Kinase**								
YOL045W	PSK2	protein serine/threonine kinase activity	37%	−12.7	0.6%	14%	−10.5	2.0%
YKL116C	PRR1	receptor signaling protein serine/threonine kinase activity	23%	−29.3	0.3%	5%	−14.8	1.1%
**Transcription**								
YOR083W	WHI5	specific transcriptional repressor activity	32%	−20.7	0.3%	22%	5.8	2.7%

**Table 2:** Yeast genes that suppress FHV replication.

Nucleus								
ORF	Gene	Molecular Function	RNA1 (%WT)	t1	q1	RNA3 (%WT)	t3	q3
**Transcription**								
YDR295C	HDA2	histone deacetylase activity	572%	5.6	1.5%	735%	4.58	3.2%
YNL059C	ARP5	structural molecule activity	564%	8.8	0.8%	924%	7.47	2.2%
YJL115W	ASF1	histone binding	413%	21.3	0.3%	604%	22.00	0.6%
YER040W	GLN3	metal ion binding	389%	10.0	0.7%	330%	3.21	4.7%
YLR226W	BUR2	cyclin-dependent protein kinase regulator activity	358%	5.8	1.5%	347%	6.20	2.6%
YML036W	CGI121	molecular_function unknown	226%	5.7	1.5%	277%	3.62	4.3%
YEL009C	GCN4	DNA binding	201%	5.7	1.5%	259%	10.02	2.0%
YDL115C	IWR1	molecular_function unknown	511%	5.2	1.6%	695%	3.97	4.0%
**Chromatin remodeling**								
YEL044W	IES6	molecular_function unknown	372%	7.3	1.0%	759%	5.12	2.9%
YJL127C	SPT10	sequence-specific DNA binding	362%	7.2	1.0%	446%	3.92	4.0%
**DNA repair**								
YDR369C	XRS2	DNA binding	283%	4.0	2.5%	326%	3.40	4.6%
**Cell Cycle**								
YAL024C	LTE1	guanyl-nucleotide exchange factor activity	453%	11.4	0.6%	873%	7.78	2.2%
YHR191C	CTF8	molecular_function unknown	416%	7.7	0.9%	477%	4.83	3.1%
YPL008W	CHL1	nucleotide binding	289%	20.0	0.3%	448%	5.58	2.8%
^#^YPR046W	MCM16	protein binding	276%	7.0	1.1%	389%	3.13	4.8%
YMR014W	BUD22	molecular_function unknown	566%	8.8	0.8%	901%	6.20	2.6%
**Cytoplasmic**								
**ORF**	**Gene**	**Molecular Function**	**RNA1 (%WT)**	**t1**	**q1**	**RNA3 (%WT)**	**t3**	**q3**
**Translation, protein biosynthesis & ribosome biogenesis**								
YDR025W	RPS11A	structural constituent of ribosome	677%	5.2	1.6%	637%	3.85	4.0%
YLR441C	RPS1A	structural constituent of ribosome	593%	5.8	1.5%	1043%	3.74	4.1%
YKL006W	RPL14A	structural constituent of ribosome	585%	17.5	0.4%	691%	7.90	2.2%
YGR027C	RPS25A	structural constituent of ribosome	484%	6.9	1.1%	584%	5.09	2.9%
*YGL076C	RPL7A	structural constituent of ribosome	431%	9.0	0.8%	628%	5.96	2.7%
YNL069C	RPL16B	structural constituent of ribosome	342%	21.7	0.3%	661%	10.11	2.0%
YMR142C	RPL13B	structural constituent of ribosome	310%	7.6	0.9%	414%	4.24	3.6%
YMR143W	RPS16A	structural constituent of ribosome	299%	7.7	0.9%	306%	5.19	2.9%
YMR230W	RPS10B	structural constituent of ribosome	239%	4.2	2.3%	293%	4.35	3.6%
YPR132W	RPS23B	structural constituent of ribosome	229%	6.6	1.2%	332%	9.30	2.2%
YDL083C	RPS16B	structural constituent of ribosome	212%	13.5	0.6%	333%	3.92	4.0%
YBL072C	RPS8A	structural constituent of ribosome	209%	7.6	0.9%	322%	7.43	2.2%
**RNA processing and stablity**								
YOL041C	NOP12	RNA binding	244%	7.0	1.1%	364%	3.11	4.9%
YGR159C	NSR1	nucleotide binding	571%	11.1	0.6%	644%	5.05	2.9%
YKL009W	MRT4	mRNA turn over and ribosome assmebly	558%	3.2	3.8%	853%	3.05	5.0%
^#^YOR076C	SKI7	3′-5′ exonuclease activity	309%	17.2	0.4%	343%	5.47	2.8%
^#^YLR398C	SKI2	RNA helicase activity	335%	7.6	0.9%	339%	5.84	2.7%
^#^YPR189W	SKI3	translation repressor activity	254%	8.7	0.8%	324%	5.53	2.8%
YHR081W	LRP1	nuclear exosome (RNase complex)	336%	11.9	0.6%	433%	7.28	2.2%
YPL213W	LEA1	RNA splicing factor activity, transesterification mechanism	887%	3.9	2.6%	1119%	3.47	4.5%
^#^YCR063W	BUD31	RNA splicing	194%	3.0	4.5%	152%	3.32	4.7%
YPL157W	TGS1	transferase activity	584%	19.2	0.3%	981%	4.42	3.5%
**Transport**								
^#^YLR373C	VID22	vacuola transport	381%	9.5	0.7%	569%	4.89	3.0%
YPR139C	VPS66	vacuola protein sorting	380%	6.5	1.2%	741%	4.32	3.6%
* ^#^YKL041W	VPS24	protein binding	369%	5.1	1.7%	585%	3.77	4.1%
YLR360W	VPS38	late endosome to vacuola transport	340%	10.4	0.7%	461%	7.53	2.2%
YGL023C	PIB2	metal ion binding	312%	5.4	1.6%	283%	4.17	3.6%
YDR320C	SWA2	protein binding	505%	8.3	0.9%	651%	6.47	2.6%
YPL195W	APL5	protein binding	220%	8.2	0.9%	246%	17.99	0.7%
YDR276C	PMP3	cation transport	166%	7.3	1.0%	204%	11.25	2.0%
^#^YPL226W	NEW1	nucleotide binding, ATPase	200%	7.9	0.9%	214%	3.37	4.6%
**Chaperone**								
YJR032W	CPR7	chaperone activity	320%	6.4	1.3%	438%	3.23	4.7%
**Metabolism**								
YDR226W	ADK1	nucleotide binding	430%	6.1	1.4%	663%	5.86	2.7%
YML022W	APT1	transferase activity	208%	3.5	3.3%	267%	5.09	2.9%
YPL017C	IRC15	oxidoreductase activity	238%	11.3	0.6%	386%	7.90	2.2%
YHR204W	MNL1	hydrolase activity	299%	5.0	1.7%	313%	5.08	2.9%
**Protein modification & signal transduction**								
YOR080W	DIA2	SCF ubiquitin ligase complex	277%	4.4	2.2%	575%	4.22	3.6%
YMR116C	ASC1	signal transducer activity	593%	10.8	0.6%	760%	5.17	2.9%
**Unknown**								
YOR235W	IRC13		427%	10.2	0.7%	613%	6.563	2.6%
YLR232W			302%	9.2	0.7%	314%	7.875	2.2%
^#^YDR241W	BUD26	bud site selection	515%	12.6	0.6%	981%	8.393	2.2%

#Genes also identified to affect BMV replication.

*Genes also identified to affect TBSV replication.

### Diverse Functions of Genes Validated to Affect FHV Replication

Among the 65 confirmed genes, only eight showed decreased FHV replication when deleted, indicating that their functions support FHV replication (Table 1 and Figure 3A). Three of these genes encode proteins that localize to mitochondria, the sites of FHV RNA replication [10], [18]. These include one mitochondrial ribosomal protein, *YPL183W-A*, and two subunits of the succinate dehydrogenase complex, *SDH1* and *SDH5* (also named *EMI5*). The remaining 5 genes encode cytoplasmic proteins, including the *PRR1* and *PSK2* kinases involved in signal transduction [19], [20]; inositol phosphate kinase *VIP1* that functions in actin cytoskeleton organization [21]; *EGD1*, a ribosome-associated chaperone involved in folding and targeting of newly synthesized peptides, including some destined for mitochondria [22]; and *WHI5,* a cell cycle-regulated transcriptional repressor that accelerates G1/S transition [23].

Fifty-seven confirmed genes showed increased levels of FHV replication when deleted, indicating that these genes normally repress FHV replication (Table 2 and Figure 3B). For some deletion strains in this class, the mean levels of viral RNA1 and RNA3 replication were associated with relatively large standard deviations, but nevertheless passed statistical confirmation due to variably but consistently increased RNA accumulation. For example, in yeast with a deletion of *LEA1*, FHV RNA1 accumulation in individual experiments was always increased above wt yeast, but ranged from 2.6- to 23-fold higher than in wt yeast (Figure 3B).

Among the 57 confirmed genes whose deletion increased FHV RNA replication, 41 encoded proteins that localized to the cytoplasm, while 16 encoded proteins that normally localized to the nucleus (Table 2). Of the 41 cytoplasmic proteins, the largest functional group comprised 12 genes encoding ribosomal proteins, which is the only functional group of genes that was over-represented (p value = 8.3E-06) by GeneOntology pathway analysis (Table 2). 10 of those genes are involved in RNA processing and stability. Their functions in RNA degradation may explain their roles in suppressing FHV RNA accumulation. Nine genes function in protein trafficking and vesicle mediated transport between sub-cellular organelles, which may suppress FHV replication by modulating trafficking of required viral proteins, cellular proteins or membrane lipids. The remaining implicated genes encode a stress response chaperone *(CPR7)* and proteins involved in nucleoside metabolism (*ADK1, APT1*), other metabolic processes (*IRC15, MNL1*), protein ubiquitination (*DIA2*), signal transduction (*ASC1*), and unknown functions (*YOR235W, YLR232W*, *YDR241W*).

The 16 genes with products normally localized to the nucleus include eight genes that function in transcription, two in chromatin remodeling, one in DNA repair and five in the cell cycle (Table 2). Since FHV RNA replication is not known to require any nuclear stage, these genes might function indirectly in FHV replication in yeast, as in affecting expression of more directly acting host factor(s). Also, since DNA expression plasmids were used to initiate *trans* (pFA + pF1R) and *cis* (pF1) FHV RNA replication systems for primary genome-wide screening and validation testing, these genes might also affect plasmid-directed transcription or nucleo-cytoplasmic export of viral RNA.

### Distinct Interactions of FHV and Other Viruses with Yeast YKO Strain Library

The yeast YKO system has previously been used to study host factor interactions of two other viruses, brome mosaic virus (BMV) [11] and tomato bushy stunt virus (TBSV) [12]. The FHV, BMV and TBSV studies were similar in that all three targets are positive strand RNA viruses, all three studies used the same YKO library, and the BMV and FHV screens both applied whole cell assays of *Renilla* luciferase expressed through viral RNA replication and sub-genomic mRNA synthesis. Interestingly, however, few host genes were identified in common between these studies. Only one gene, e.g., was identified to affect all three viruses: *VPS24,* a subunit of endosomal sorting complex required for transport III (ESCRT-III), whose deletion decreased TBSV and BMV replication, but increased FHV replication. The only additional common genes revealed by pairwise comparisons were ribosomal protein gene *RPL7A* (Table 2 ORF labeled with *), whose deletion increased both FHV and TBSV replication, and nine genes (14% of host genes validated to affect FHV) that affected both BMV and FHV.

**Figure 4:**
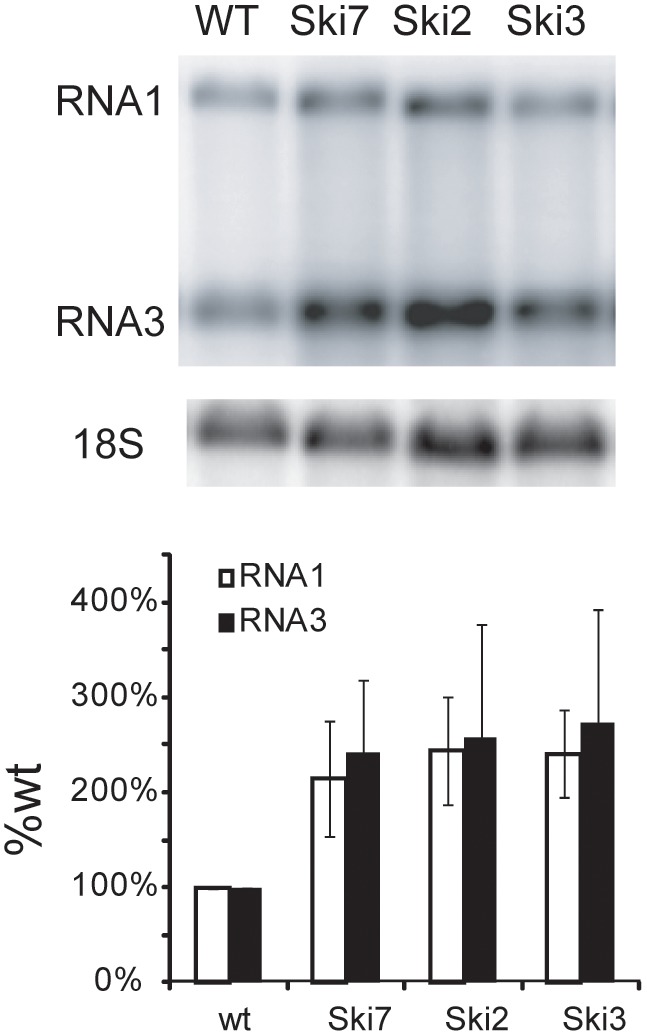
Genes in the *Ski* pathway normally suppress FHV replication in yeast. Yeast strains with the indicated *Ski* gene deletions were transformed with plasmid pF1 (Figure 1A) and, following galactose-induction of pF1 transcription, FHV genomic RNA1 replication and subgenomic RNA3 production were assayed by Northern blot hybridization. The histograms show the averages and standard deviations of RNA1 and RNA3 levels across four independent experiments. Representative Northern blots are shown above the histograms.

Among five host genes with similar effects on BMV and FHV replication, three are related RNA degradation genes *Ski2*, *Ski3/5*, and *Ski7,* which repress the levels of endogenous dsRNA replicons in yeast [24]. Similarly, deleting any of these genes increased accumulation of both FHV and BMV RNA replication products in yeast by 2–3 fold (Figure 4A and reference [11]). Both FHV and BMV replication also were suppressed by *BUD31*, an RNA splicing factor [25] and *VID22*, implicated in membrane import and DNA repair [26].

Three genes that facilitate BMV replication were found here to suppress FHV replication (Table 2 ORFs labeled with #). These include *NEW1*, involved in mRNA export and ribosome biogenesis [27]; *MCM16*, which functions in chromosome segregation [28]; and *BUD26*, a gene of unknown function. In addition to these whole genome screening results, our recent targeted studies [29] revealed that RNA processing genes *Lsm1*, *Lsm6*, *Lsm7,* and *Dhh1* are required to efficiently recruit BMV genomic RNAs into both translation and RNA replication [30], but restrict FHV RNA accumulation [29], possibly through their roles in deadenylation-dependent mRNA-decapping and decay. Additionally, 14 genes implicated in BMV replication [11] were excluded from the present FHV study due to growth defects on solid or liquid media after their knockout strains were transformed with pFA and pF1R (see table S1).Similarly, one gene implicated in TBSV replication [12] was excluded for the same reason.

The low overlap among host genes implicated in modulating FHV, BMV and TBSV replication is not surprising because many aspects of the biology of these viruses are distinct. For example, FHV naturally infects insects and replicates its RNA on the outer membranes of host cell mitochondria [10], while BMV and TBSV infect different plants and form RNA replication complexes on endoplasmic reticulum (ER) and peroxisome membranes, respectively [8], [31]. Moreover, while FHV encodes only the single replication protein, protein A (Figure 1), BMV and TBSV each encode two replication proteins, an RNA-dependent RNA polymerase and an essential auxiliary protein that directs membrane targeting and modification [32], and for BMV this auxiliary protein contains an RNA helicase-like domain that FHV and TBSV lack [33].

In addition to low overlap at the gene specific level, the FHV, BMV and TBSV YKO screens differed in the balance of virus-supporting and –interfering host genes identified. For FHV, 57 of 65 confirmed host genes (88%) increase virus replication when deleted, in contrast to TBSV, for which 90 of 96 confirmed genes (94%) decreased virus replication when deleted [12]. In contrast to these extremes, the BMV screen returned a more even result, with 39 of 97 confirmed host genes (40%) increasing and 58 genes (60%) decreasing BMV replication when deleted. The low yield of host genes that facilitate FHV replication may be related to the gene knockout screening approach used and FHV’s ability to perform its intracellular replication steps in an unusually broad range of host cells, including insect [9], [18], mammalian[34], plant [35], [36] and yeast [4], [13] cells. Given this broad host competence, whatever host genes are required for FHV replication must be conserved across kingdoms, and therefore are more likely to be essential for cell growth and thus excluded from the YKO non-essential gene library studied here.

In keeping with the distinct groups of host genes required by these three positive-strand RNA viruses, individual retroviruses like HIV-1 and Moloney murine leukemia virus (MuLV) also each require or are restricted by many different host factors [37], [38]. Overall, recent genome-wide studies have revealed that viruses depend on an amazing variety of host genes for most if not all of their functions [39]–[43]. The continuing elucidation of such virus-host interactions promises to radically transform understanding and control of viruses.

## Materials and Methods

### Plasmid Construction

pFA was generated by cloning the *HindIII/SpeI* fragment of pBDL7[13] into pRS425 [44]. pF1R was generated by replacing the GFP ORF in pF1_fs_-GFP_N2_ [4] with the *Renilla* luciferase ORF downstream of FHV B1 AUG codon.

### Yeast Transformation, Culturing, and *Renilla* Luciferase Assays

Yeast strain BY4743 (WT) and the homozygous diploid deletion series (BY4743 strain background) were obtained from Research Genetics (Huntsville, AL). Standard yeast techniques were used. 96-well transformation and the *Renilla* luciferase assay were performed as previously described [11]. In brief, each yeast deletion mutant was inoculated in 0.5 ml YPAD plus glucose medium in a separate well of a 96-well high capacity plate, and grown at 30°C overnight. To increase transformation efficiency, the overnight culture was sub-cultured in YPAD plus glucose at ratio of 1∶10 and grown four hours at 30°C. The yeast cells then were pelleted and resuspended in 100 µl PEG/LiAc transformation solution containing 1 µg of each plasmid. The plate was incubated at 30°C for one hour, heat-shocked at 42°C for 15 minutes, and 5 µl of each transformation mixture was plated on synthetic defined (SD) medium with appropriate amino acids omitted to select for all desired plasmids. After three days incubation at 30°C, yeast transformants were ready for further analysis.

To assay for FHV directed replication and expression of the *Renilla* luciferase reporter gene as in Fig. 1B, yeast strains were transformed with pFA and pF1R plasmids as described above, and the resulting yeast transformants were inoculated into liquid SD-medium containing glucose, and grown at 26°C for one day. This and all subsequent media used with these transformants had histidine and leucine omitted to select for pFA and pF1R. The yeast culture was then sub-cultured into SD-medium containing galactose at a starting OD_600_ = 0.075, grown for one day at 26°C, and sub-cultured again in galactose-SD-medium at OD_600_ = 0.075 and grown at 26°C overnight. This final overnight culture was assayed as previously described [11] for FHV replication-dependent expression of *Renilla* luciferase activity.

### Data Processing

The *Renilla* luciferase activity of each deletion strain in relative light units (rlu) was transformed by taking the 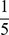 root to stabilize the variability. The measurement of each yeast strain was normalized to the median measurement of the plate containing that strain. The OD_600_ numbers of all strains were measured to monitor the growth of yeasts, and were normalized to the plate median as well. 5% of the total strains that grew too fast and 5% that grew too slow were removed from the final analysis.

### Validation Testing of Yeast Strains

For validation testing, selected candidate yeast strains were grown in 96-deep-well plates and transformed as described above with pF1. Four colonies of each transformed strain were picked to check for FHV replication. The yeast were again grown in glucose-SD-medium for one day, then sub-cultured twice in galactose-SD-medium as described above for primary screening. Total yeast RNA was isolated [45]. Accumulation of FHV RNAs and 18S rRNA as a loading control were assayed by Northern blot analysis [11]. The RNA signals were quantitated using the Quantity1 (Bio-Rad) software package. Statistical analysis of RNA levels was performed as previously described [46]. FHV RNA1 and RNA3 levels were normalized to the 18S rRNA level in the same sample.

Using the R statistical package (version R-2.11.1) (http://www.r-project.org/), one sided T-tests were performed to identify mutant yeast strains with significantly altered FHV replication relative to wild type yeast. Q-values were calculated based on t-scores to control the false discovery rate under 5% [47].

## Supporting Information

Table S1 Whole screen data for FHV replication in yeast knockout library (YKO).
(XLSX)
